# Virtual Screening of IL-6 Inhibitors for Idiopathic Arthritis

**DOI:** 10.6026/97320630015121

**Published:** 2019-02-28

**Authors:** Palak Shukla, Ravina Khandelwal, Diksha Sharma, Anindya Dhar, Anuraj Nayarisseri, Sanjeev Kumar Singh

**Affiliations:** 1 In silico Research Laboratory, Eminent Biosciences, Mahalakshmi Nagar,Indore – 452010,Madhya Pradesh, India; 2 Bioinformatics Research Laboratory, LeGene Biosciences Pvt Ltd.,Mahalakshmi Nagar,Indore - 452010,Madhya Pradesh,India; 3 Computer Aided Drug Designing and Molecular Modelling Lab,Department of Bioinformatics, Alagappa University, Karaikudi-630 003, Tamil Nadu, India

**Keywords:** IL-6, Idiopathic Arthritis, Molecular Docking, Virtual Screening

## Abstract

Juvenile idiopathic arthritis (JIA) is a heterogeneous disease characterized by the arthritis of unknown origin and IL6 is a known target for JIA. 20 known inhibitors towards IL-6 were screened and Methotrexate (MTX) having PubChem ID: 126941 showed high binding capacity with the receptor IL-6. The similarity searching with this compound gave 269 virtual screened compounds. The said screening presented 269 possible drugs having structural similarity to Methotrexate. The docking studies of the screened drugs separated the compound having PubChem CID: 122677576 (re-rank value of -140.262). Toxicity and interaction profile validated this compound for having a better affinity with the target protein. Conclusively, this study shows that according to ADMET profile and BOILED-Egg plot, the compound (PubChem CID: 122677576) obtained from Virtual Screen could be the best drug in future during the prevention of juvenile idiopathic arthritis. In the current study, the drug CID: 122677576 is a potent candidate for treating JIA. The pharmacophore study revealed that the drug CID: 122677576 is a non-inhibitor of CYP450 microsomal enzymes and was found to be non-toxic, similar to the established drug Methotrexate (CID: 126941). It has a lower LD50 value of 2.6698mol/kg as compared to the established compound having LD50 value as 23.4955mol/kg. Moreover, the compound was found to be non-carcinogenic.

## Background

Juvenile idiopathic arthritis is a group of diseases characterized by arthritis of unknown origin, which is seen before age of 16 years [1]. It is a group of diseases that encompasses all forms of arthritis beginning before the age of 16 years and stays for more than 6 weeks. 250,000 children in the United States alone are estimated to be affected by Juvenile Idiopathic Arthritis (JIA). Short and long term disabilities are also caused by JIA. There are three major types of presentation of the term JIA, which encompasses a heterogeneous group of diseases: (a) oligo-arthritis, (b) polyarthritis and (c) systemic-onset JIA (SoJIA) [2]. One subspace, systemic JIA (sJIA), is the additional presence of exhausting fever, serositis (inflammation of the serous tissues of the body), fugitive rash, lymph adenopathy (disease of the lymph nodes, in which they are abnormal in size) and hepatosplenomegaly (a disorder where both the liver and spleen swell beyond their normal size). Osteoporosis, growth retardation, systemic amyloidosis and macrophage activation syndrome are some severe complications. These are observed more frequently in patients with the longstanding disease than in other JIA subclasses. Patients of sJIA have a range of other prominent features, which includes marked elevation of erythrocyte sedimentation rate (ESR) and C-reactive complement protein (CRP), leucocytes with high neutrophil counts and thrombocytosis. It has a yearly occurence of 2-20 cases per 100,000 population in countries having a high income.

IL-6 has a pleiotropic effect on inflammation, immune response, and hematopoiesis. It is a soluble mediator. Human IL-6 is composed of 212 amino acids, including a 28-amino-acid signal peptide. Its gene has been mapped to chromosome 7p21. Although the core protein is about 20kDa, glycosylation accounts for the size of 21-26 kDa of natural IL-6. Disproportioned continual synthesis of IL-6 has a pathological impact on chronic inflammation and autoimmunity. A humanized anti-IL-6 receptor Tocilizumab is an antibody was developed for the same reason [3]. It is evident that the production of IL-6 is particularly high in sJIA. By a variant of the gene encoding IL-6 in a significant fraction of patients, it can be genetically determined. A significant increase in soluble IL-6 receptor (sIL-6R) concentrations, in addition to the increase in serum IL-6, has been determined in the JIA patients. Different levels of IL-6 correlate with the activity of disease, pattern of fever and platelet counts, which indicates an important role of IL-6 in the pathogenesis of sJIA. A humanized anti-human IL-6 receptor antibody MRA (Tocilizumab) of kappa-IgG1 subclass is developed collaboratively by Osaka University and Chugai Pharmaceutical Company Ltd (Japan). MRA is humanized as the complementary determining regions of a mouse anti-human IL-6 receptor monoclonal antibody are grafted onto human IgG1 by using recombinant DNA technology [3].

## Methodology

### Selection of inhibitors

For the determination of inhibitors in the present examination, the existing inhibitors of IL-6 against Juvenile Idiopathic Arthritis were chosen from various literature studies. The accessibility of preexisting inhibitors is 20, chosen to promote perceptions (Table1).

### Protein and Ligand preparation:

**Figure 1:**
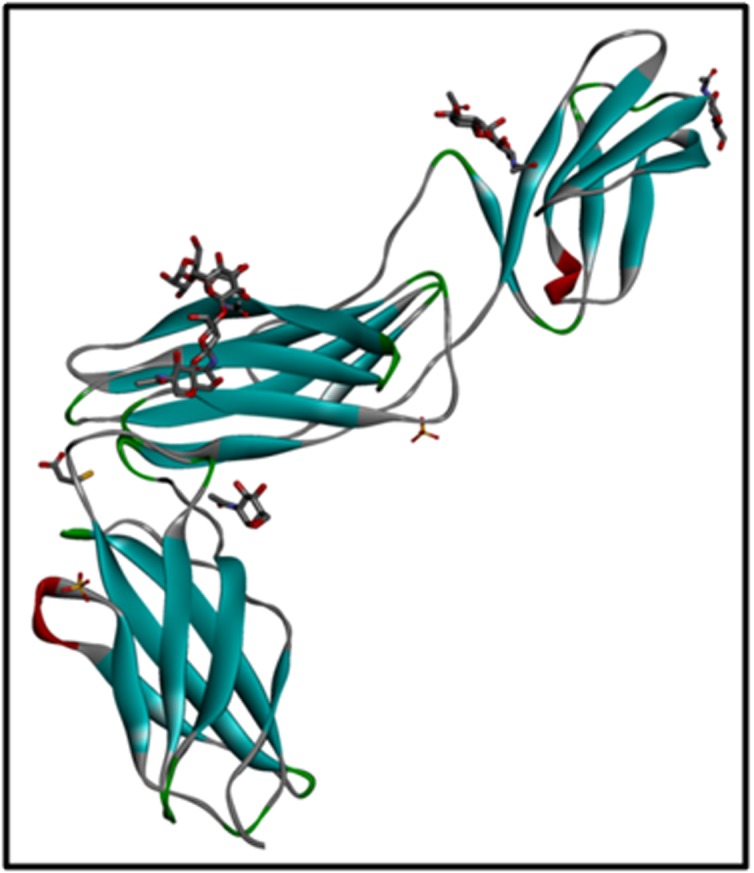
Protein 3D structure of IL-6 obtained from PDB (PDBID: 1N26) Visualization in Accelrys Discovery Studio

The crystal structure of target protein, extracellular domain of IL-6 was retrieved from Protein Data Bank (PDB) with PDB ID: 1N26 [6] and was carried further for more studies of docking process (Figure 1). The inhibitors accomplishing a PubChem CID have redeemed the 3D conformer of inhibitors and saved in SDF format [7-11]. Further, preparation of ligands was preceded by taking the 3D structure of all those compounds inserted in LegPrep module of Schrodinger suite, 2013 (Schrodinger. LLC, New York, NY) and were optimized through OPLS 2005 force field algorithm [13-18]. The prepared ligands were saved in a single SDF file for further docking studies [19-23].

The crystal structure of target protein, additional extracellular province of IL-6 was recovered from Protein Data Bank (PDB) with PDB ID: 1N26 and was sent for additional investigations of the docking process. The inhibitors achieving a PubChem ID redeemed the 3D conformer of inhibitors and spared as SDF design. Certain compounds lacked PubChem CID and 3D structures. Marvin Sketch was used to make the 3D structures of such compound and was allowed in SDF design [24-27]. Assist readiness of ligand was done before taking the 3D structure of each one of those compounds installed in LigPrep module of Schrodinger suite, 2013 (Schrodinger. LLC, New York, NY) and were advanced through OPLS 2005 power field calculation [28-32]. The ligands, which were ready, spared in single SDF petition for additionally docking investigations.

### Molecular docking:

Molegro Virtual Docker (MVD) was used for the molecular docking studies, which was unified with high potential Piece-Wise Linear Potential (PLP) and MolDock scoring function [33-36]. All the preprepared 20 ligands were saved in one single SDF file. The PDB file of target protein consist pre-existing ligands, which were removed and prepared by detecting cavities, and those which were found in the first cavity, bear the highest volume were targeted for the further procedure of docking with ligands. Docking process possessing parameter of maximum reiteration of 1500, maximum population size 50, Grid solution 0.2 having a binding affinity, the protein and ligands were evaluated on the following confirmation of the Internal Electrostatic interaction (Internal ES), sp2-sp2 torsions and internal hydrogen bond interaction. The binding site is defined as the first cavity possessing high volume. A post dock study involves energy minimization and H-bond optimization. Setting of Simplex Evolution at max steps 300 and neighbor distance faster 1.00 [37-39]. After docking, to minimize the complex energy of ligand-receptor interaction the Nelder Mead Simplex Minimization (using non-grid force field and H-bond directionality) was used [40-42].

### Virtual screening:

Using Methotrexate as a query compound, the present investigations were performed for the structure similarity search analysis from the PubChem database, which is maintained by NLM (National Library of Medicine, NCBI, NIH). The filtration properties parameter is set by the component rule of Lipinski's rule of five at Threshold >=95% against NCBI's PubChem database [43-45].

### Drug-Drug comparative study:

The unnamed complex structure was redeemed from an established drug docking result and was simply imported. It was cleaned by removing all ligand constraints and eventually imported the bestposed drug and exported as best drug docked file in SDF format. Again the complex structure was retrieved from virtual docking result and the procedure was repeated. The excel sheet was prepared to check all the affinities, hydrogen interaction and high re-rank score. An excel sheet was prepared in order to identify the best drug [46-47].

### Pharmacophore studies:

Pharmacophore studies involve different types of interactions between ligands and receptors. It includes H-bond interactions, electrostatic interactions, hydrophobic interactions, and aromatic interactions. The study is done using Accelrys Discovery Studio 3.5 DS Visualizer [46-48].

### ADMET studies:

Owing to the superior affinity of best docked established compound Methotrexate (CID: 126941) and virtual screened compound PubChem CID-122677576, the bioactivity properties, and toxicity was predicted by using admetSAR [7-9].

### Software, Suites and Web servers Used:

NCBI's PubChem was used to retrieve all the chemical 3D structures in SDF format. The ligands were optimized by using the software Schrodinger suite 2013 (Schrodinger.LLC, 2009, New York, NY). Flexible Docking was performed by making target and all the compounds in Molegro Virtual Docker 2010.4.0.0. Molecular Visualization was done with Accelrys Discovery Studio® Visualizer 3.5.0.12158 (Copyright© 2005-12, Accelrys Software Inc.). ADMET profiles were studied and calculated using admetSAR (Laboratory of Molecular Modelling and Design © 2012 East China University of Science and Technology, Shanghai Key Laboratory for New Drug-Drug Design).

## Results and Discussion

The docking studies of complete pre-established 20 drugs were performed and it was found that the compound Methotrexate (CID: 126941) is the best-established compound. Table 2 as the compound having lowest energy with -105.677 as re-rank score shows the higher affinity score directed towards our target protein and has the great affinity properties like molecular weight 454.447 g/mol, 5 hydrogen bond donor and 12 hydrogen bond acceptor, topological polar surface area 211 A^2 and log value of-1.8.Thus, the compound reveals the superior inhibitory affinity over protein IL-6.The docking studies were resulted in Table 2.

Further, the similarity search for this inhibitor displayed 269 compounds. Table 3 contains the docking result of the top 10 of 269 virtual screened compounds. The compound with PubChem CID122677576 with higher affinity is selected. This compound has a molecular weight of 496.528 g/mol, 5 hydrogen bond donor and 12 hydrogen bond acceptor, a topological surface area of 208 A^2and a log P value is -0.7. Similarly, among all 269virtual screened compounds and 20 pre-established compounds, the drug with PubChem CID: 122677576 have much potential inhibition against juvenile idiopathic arthritis over the target protein IL-6.

The compound with PubChem Id: 122677576 prove more efficient than the already established drug Methotrexate and it is shown in Table 4. External ligand interactions and Protein-ligand interactions, along with total energy evidently shows the stable interactions of the compound (PDB ID: 122677576) with the target protein IL-6. Moreover, steric energy for the established compound also indicates the better stability of the compound obtained after the virtual screening studies.

Pharmacophore study is done for the better clarification of the interactive attributes of the compound, which are important for the biological functioning of that compound. The pharmacophore mapping gives spatial essential systematic features of the molecular interaction with a specific target receptor apart from the method of molecular docking. Pharmacophore studies provide accurate query on the optimum interaction with suitable target annotations and represent the aligned poses of the molecule and help us to find the high interaction mode between target protein and compound. Owing to admirable affinity and good interaction profile of virtual screened compound (PubChemCID 122677576) over the most effective pre-established compound Methotrexate (PubChem CID 126941), the study carries forwarded to the pharmacophore results. Pharmacophore mapping results in the positive intensities of electrostatics as well as varying intensities and the charges in aromatic interaction, respectively. The pharmacophoric feature includes the study of different types of interactions, such as H-bond interactions, electrostatic interactions, hydrophobic interactions, aromatic interactions, and van der Waals (vdW) interactions. These interactions are shown below.

**Figure 2:**
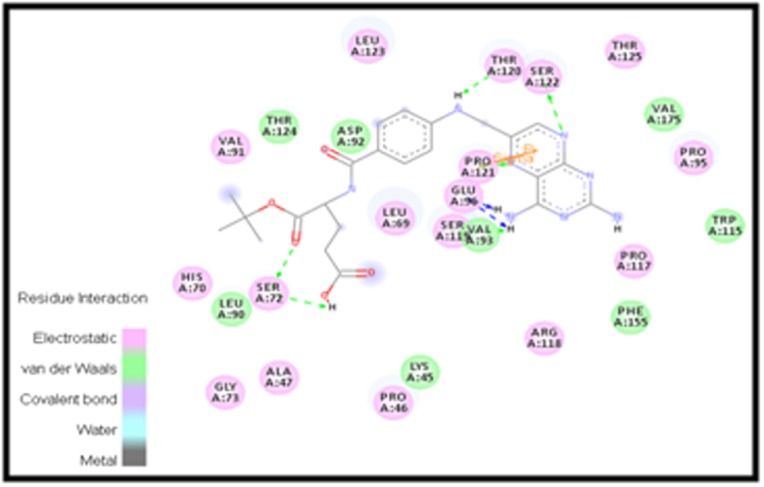
The most effective compound (PubChem id: 122677576) binding with IL-6 obtained from the virtual screening studies shows Van der Waals Interaction

Figure 2 represents Van der Waals interaction between residues interactions of virtual screened compound (PubChem CID- 122677576) present in the cavity of IL-6 protein. The interaction in the figure represents the residues with ligands displayed in green to be van der Waals interaction and the residues displayed in pink to be electrostatic interactions. The figure depicts four hydrogen bond interaction; two with Ser72, one with Thr120 and Ser122 residues represented by a green dotted line. Consequently, the van der Waal interactions were also shown by the residues Thr124, Asp92, Leu90, Lys45, Val93, Phe155, Trp115 and Val175 were circled with green. Furthermore, the interaction of pi-pi between the Pro121 and the compound are depicted in orange color.

**Figure 3:**
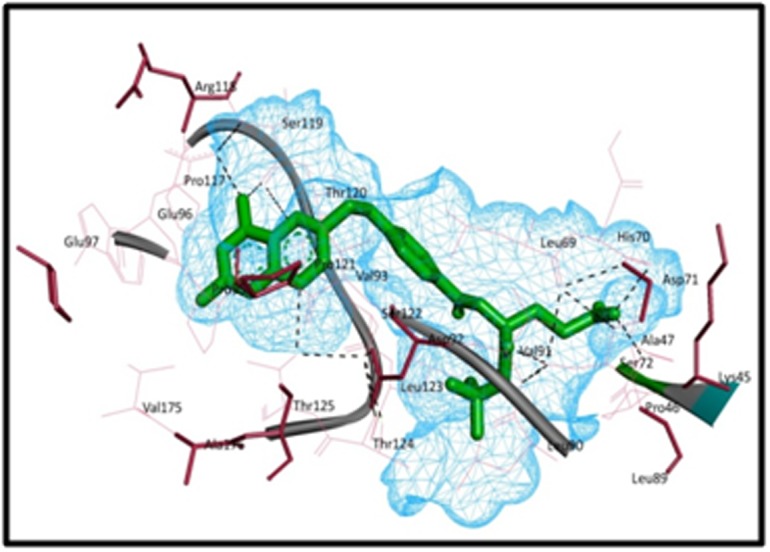
The most effective compound (PubChem id: 122677576) binding with IL-6 obtained from the virtual screening studies shows Ligand-receptor Interaction

Figure 3 represents the receptor-ligand binding, by producing a signal by binding to a site on targeted IL-6 protein resulted in the change in conformation of IL-6 protein according to the ligand. In the figure receptor-ligand interaction of the most effective virtual screened compound (PubChem CID-122677576) with different amino acid residues present in the ligand. The figureshows the ligand-receptor interactions depicted by a black dotted line with residues Asp71, Ser72, Val91, Ser119, Pro121, Ser122, Thr124, Thr125, Thr120, Pro117 and His70. This interaction shows the high affinity of the virtual screened compound in comparison with best pre-established compound Methotrexate (PubCID-126941) having the lowest re-rank score.

## ADMET profile:

Table 5 is the ADMET prediction of both the best-docked compound Methotrexate (PubChem CID 126941) and the best virtual screened compound (PubChem CID - 122677576). According to the table, brain penetration prediction i.e.Blood-Brain Barrier (BBB), Methotrexate and virtual screened compound (PubChem CID 122677576) are showing the negative value to the property of absorbing. Human Intestinal Absorption (HIA) shows the greater absorption in the intestine and both the compounds denote equal parameter. For the predictions of P-glycoprotein substrate and Pglycoprotein inhibitor, both the compounds show alternative similarity. At the absorption site of the P-glycoprotein Substrate, both the compounds show exactly the same probability while Pglycoprotein Inhibitor shows the values with high probability. In addition to the distribution of sub-cellular localization, both the compounds are localized in the mitochondria. The mitochondrial distribution of both compounds shows a distribution that is almost the same to each other. In case of metabolism, both the compounds are acting as the substrates as well as the inhibitors. The compounds display equivalent high inhibitory effect towards the target protein. The further study of bioactivity in the profile of excretion and toxicity is almost equivalent. In reference to carcinogens, they both show the same carcinogenicity. The mutagenicity of the compound can be predicted by ADMET regression toxicity study. Both the compounds in the properties of Rat Acute Toxicity are nearly equal to each other. The possibility of having higher toxicity than these two molecules is shown in Table 6. Further, the study of bioactivity in the profile of excretion and toxicity is similar.

## Comparative ADMET profile study of the compounds and the control:

**Figure 4:**
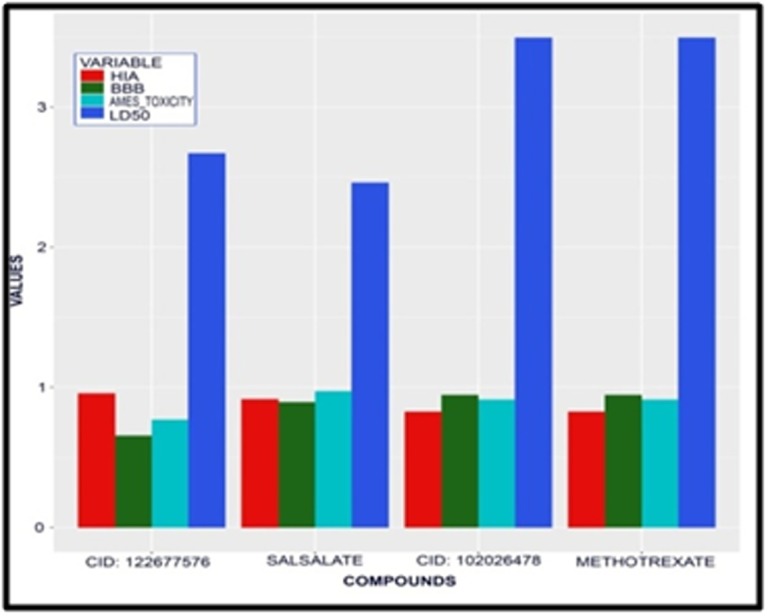
Comparative ADMET studies of BBB, HIA, AMES toxicity and LD50 of the Established Compounds against Virtual screened compounds

The comparative ADMET profile for the inhibitors was predicted based on the parameters such as Blood-Brain Barrier (BBB), Human Intestinal Absorption (HIA), AMES Toxicity and LD50. The established compound Methotrexate with PubChem CID:126941 and the best virtual screened compound with PubChem CID- 122677576 along with other top 2 compounds Salsalate having PubChemCID5161 and the compound having PubChemCID102026478 was preferred for comparative ADMET studies. These four compounds were graphically estimated using R-programming as shown in Figure 4. The parameters: BBB, HIA, AMES toxicity and LD50 procured from the admetSAR database. The compounds were tabulated according to their predicted values and properties. So, according to the graph as well as Table 7 among all the four compounds the values of BBB are similar in both the compound Methotrexate (PubChem CID 126941) and the compound (PubChem ID-102026478). Human Intestinal Absorption (HIA) value is similar in both the compound Methotrexate (PubChem CID 126941) and the compound (PubChem ID- 102026478). LD50 in rat is lower in Salsalate (PubChem ID-5161). The compound with PubChem ID-122677576 has higher HIA. Methotrexate has an equivalent property in LD50 in rat and Ames toxicity in comparison with compound (PubChem Id - 102026478), which also shows the regression in toxicity.

## Conclusion

Inhibition of IL-6 interactions has now surfaced as an important drug target against Juvenile Idiopathic Arthritis. We study 20 preestablished inhibitors of IL-6 which are associated with JIA using molecular docking analysis, an inhibitor of IL-6 for JIA among presently effective inhibitor Methotrexate and virtual screened compound Pub CID: 122677576. We foresee Methotrexate and CID: 122677576 are structurally cognant. However, Methotrexate is a good inhibitor, but compound 122677576 has the lowest re-rank score and can emerge as an important drug in the treatment of disease in the future ahead.

## Conflict of Interest

The authors declare no conflict of interest.

## Figures and Tables

**Table 1:** List of Established inhibitors collected from various literature

S. No.	Inhibitors	Pub ID	MW in gm/mol		HBD	HBA	Logp	Ref
1	Cyclosporine A	5284373	1202.635		5	12	7.5	[4]
2	Chloroxine	2722	214.045		1	2	3.5	[5]
3	Salazosulfapyridine	5359476	398.393		3	9	2.3	[5]
4	Methotrexate (MTX)	126941	454.447		5	12	-1.8	[5]
5	Aspirin	2244	180.159		1	4	1.2	[5]
6	celecoxib (Celebrex)	2662	381.373		1	7	3.4	[5]
7	Diclofenac	3033	296.147		2	3	4.4	[5]
8	Diflunisal (Dolobid)	3059	250.201		2	5	4.4	[5]
9	etodolac (Lodine)	3308	287.359		2	3	2.8	[5]
10	ibuprofen (Motrin, Advil)	3672	206.285		1	2	3.5	[5]
11	indomethacin (Indocin)	3715	357.79		1	4	4.3	[5]
12	Ketoprofen	3825		254.285	1	3	3.1	[5]
13	Ketorolac	3826	255.273		1	3	1.9	[5]
14	nabumetone (Relafen)	4409	228.291		0	2	3.1	[5]
15	Naproxen	156391		230.263	1	3	3.3	[5]
16	oxaprozin (Daypro)	4614	293.322		1	4	4.2	[5]
17	piroxicam (Feldene)	54676228	331.346		2	6	3.1	[5]
18	Salsalate	5161	258.229		2	5	3	[5]
19	sulindac (Clinoril)	1548887	356.411		1	5	3.4	[5]
20	tolmetin (Tolectin)	5509	257.289		1	3	2.8	[5]

**Table 2:** Docking results of Established Drugs

LIGAND	FILE NAME	MOLDOCK SCORE	RERANK SCORE	H BOND
126941	[00]126941	-165.255	-105.677	-10.8923
126941	[03]126941	-129.781	-97.6819	-2.7623
5161	[01]5161	-114.55	-90.8458	-6.49702
4614	[02]4614	-119.216	-89.5275	-1.02526
5161	[02]5161	-111.018	-88.7829	-3.65241
3715	[00]3715	-118.311	-88.6118	-5
4614	[00]4614	-123.091	-87.9868	-4.39387
2244	[01]2244	-98.6621	-87.9747	-4.68577
156391	[00]156391	-105.996	-87.7415	-4.97939
3308	[00]3308	-115.385	-87.5747	-5.3458
2662	[00]2662	-124.171	-87.5568	-1.91396

**Table 3:** Virtual Screening results

FILE NAME	MOLDOCK SCORE	RERANK SCORE	H BOND	MW
[00]122677576	-191.912	-140.262	-5.94855	496.519
[00]102026478	-217.813	-138.97	-11.4481	841.781
[00]132255100	-181.506	-135.599	-5.68666	439.428
[00]16218627	-180.136	-135.525	-7.672	440.413
[00]100968121	-192.426	-135.379	-2.42808	610.661
[00]128780	-173.361	-134.132	-6.10786	458.403
[00]10696709	-178.155	-133.094	-2.54635	500.483
[00]456144	-192.12	-132.787	-9.20054	712.667
[00]444319	-178.728	-131.774	-7.70866	455.447
[01]101755837	-199.698	-130.665	-6.95592	711.682

**Table 4:** Drug-drug comparison

Established Drug: Methotrexate	Virtual Screened Drug (PubChem id: 122677576)
Energy overview:Descriptors	MolDock Score	Rerank Score	MolDock Score	Rerank Score
Total Energy	-173.204	-111.978	-195.561	-143.154
External Ligand interactions	-185.718	-132.69	-209.335	-172.055
Protein - Ligand interactions	-185.718	-132.69	-209.335	-172.055
Steric (by PLP)	-166.874	-114.476	-199.728	-137.014
Steric (by LJ12-6)		-3.29		-27.433
Hydrogen bonds	-18.843	-14.924	-9.606	-7.608
Hydrogen bonds (no directionality)		0		0
Electrostatic (short range)	0	0	0	0
Electrostatic (long range)	0	0	0	0
Cofactor – Ligand	0	0	0	0
Steric (by PLP)	0		0	
Steric (by LJ12-6)		0		0
Hydrogen bonds	0	0	0	0
Electrostatic	0	0	0	0
Water - Ligand interactions	0	0	0	0
Internal Ligand interactions	12.513	20.712	13.774	28.9
Torsional strain	6.417	6.019	14.433	13.538
Torsional strain (sp2-sp2)		1.133		1.451
Hydrogen bonds		0		0
Steric (by PLP)	11.605	1.996	7.111	1.223
Steric (by LJ12-6)		11.565		12.688
Electrostatic	0	0	0	0
Soft Constraint Penalty	0		0	
Search Space Penalty	0		0	

**Table 5:** ADMET profile calculation of both best-docked compounds by AdmetSAR

Virtual Screened Drug	Established Drug
Model Absorption	Result	Probability	Result	Probability
Blood-Brain Barrier	BBB-	0.6563	BBB-	0.9467
Human Intestinal Absorption	HIA+	0.9575	HIA+	0.8261
Caco-2 Permeability	Caco2-	0.7248	Caco2-	0.7754
P-glycoprotein Substrate	Substrate	0.7871	Substrate	0.8172
P-glycoprotein Inhibitor	Non-inhibitor	0.6111	Non-inhibitor	0.7752
	Non-inhibitor	0.7509	Non-inhibitor	0.9879
Renal Organic Cation Transporter	Non-inhibitor	0.8994	Non-inhibitor	0.8886
Distribution				
Subcellular localization	Mitochondria	0.5355	Mitochondria	0.4349
Metabolism				
CYP450 2C9 Substrate	Non-substrate	0.8783	Non-substrate	0.85
CYP450 2D6 Substrate	Non-substrate	0.7845	Non-substrate	0.7968
CYP450 3A4 Substrate	Substrate	0.584	Substrate	0.5177
CYP450 1A2 Inhibitor	Non-inhibitor	0.7249	Non-inhibitor	0.9045
CYP450 2C9 Inhibitor	Non-inhibitor	0.7417	Non-inhibitor	0.907
CYP450 2D6 Inhibitor	Non-inhibitor	0.9051	Non-inhibitor	0.9231
CYP450 2C19 Inhibitor	Non-inhibitor	0.693	Non-inhibitor	0.9025
CYP450 3A4 Inhibitor	Non-inhibitor	0.6359	Non-inhibitor	0.8333
CYP Inhibitory Promiscuity	Low CYP Inhibitory Promiscuity	0.7285	Low CYP Inhibitory Promiscuity	0.9739
Excretion
Toxicity
Human Ether-a-go-go-Related Gene Inhibition	Weak inhibitor	0.989	Weak inhibitor	0.9564
	Non-inhibitor	0.6529	Non-inhibitor	0.6958
AMES Toxicity	Non AMES toxic	0.7703	Non AMES toxic	0.9132
Carcinogens	Non-carcinogens	0.9039	Non-carcinogens	0.9517
Fish Toxicity	Low FHMT	0.9896	Low FHMT	0.9534
Tetrahymena Pyriformis Toxicity	High TPT	0.9052	High TPT	0.7836
Honey Bee Toxicity	Low HBT	0.7618	Low HBT	0.8736
Biodegradation	Not ready biodegradable	0.989	Not ready biodegradable	0.9741
Acute Oral Toxicity	III	0.5639	II	0.731
Carcinogenicity (Three-class)	Non-required	0.6139	Non-required	0.6979

**Table 6:** ADMET profile (Regression)

Virtual Screened Drug: CID122677576	Established Drug
Model Absorption	Value	Unit	Value	Unit
Aqueous solubility	-3.643	LogS	-3.0651	LogS
Caco-2 Permeability	0.0081	LogPapp,cm/s	-0.3591	LogPapp,cm/s
Distribution
Metabolism
Excretion
Toxicity
Rat Acute Toxicity	2.6698	LD50,mol/kg	3.4955	LD50,mol/kg
Fish Toxicity	1.6179	pLC50,mg/L	1.82	pLC50,mg/L
Tetrahymena Pyriformis Toxicity	0.3464	pIGC50,ug/L	0.2833	pIGC50,ug/L

**Table 7:** Comparative ADMET profile of the test ligands and the control

Compounds	Blood-Brain Barrier (BBB+/BBB-)	Human Intestinal Absorption(HIA)	AMES toxicity	Carcinogenicity	LD50 in rat
CID 126941 (Methotrexate)	0.9467 (BBB-)	0.8261 (HIA+)	0.9132 (Non AMES Toxic)	Non- carcinogenic	3.4955
CID 5161 (Salsalate)	0.8946 (BBB+)	0.9161 (HIA+)	0.9731 (Non AMES Toxic)	Non- carcinogenic	2.4607
CID (122677576)	0.6563 (BBB-)	0.9575 (HIA+)	0.7703 (Non AMES Toxic)	Non- carcinogenic	2.6698
CID (102026478)	0.9467 (BBB-)	0.8261 (HIA+)	0.9132 (Non AMES Toxic)	Non- carcinogenic	3.4955

## References

[1] Ravelli A (2007). Lancet.

[2] Pascual V (2005). J Exp Med.

[3] Tanaka T (2014). Cold Spring Harb Perspect Biol.

[4] Martini A (2012). Autoimmun Rev.

[5] Yoshida Y (2014). Biomed Res Int.

[6] Varghese JN (2002). Proc Natl Acad Sci U S A.

[7] Majhi M (2018). Curr Top Med Chem.

[8] Vuree S (2013). J Pharm Res.

[9] Sharma K (2018). Curr Top Med Chem.

[10] Pandey N (2013). J Pharm Res.

[11] Sinha K (2018). Curr Top Med Chem.

[12] Shameer K (2017). Curr Neuro pharmacol.

[13] Nayarisseri A (2018). Curr Top Med Chem.

[14] Khandelwal R (2018). Curr Top Med Chem.

[15] Bandaru S (2017). PLoS One.

[16] Sharda S (2017). Curr Top Med Chem.

[17] Bandaru S (2017). Curr Drug Metab.

[18] Khandekar N (2016). Bioinformation.

[19] Basak SC (2016). Curr Pharm Des.

[20] Basak SC (2016). Curr Pharm Des.

[21] Bandaru S (2016). Gene.

[22] Natchimuthu V (2016). Comput Biol Chem.

[23] Patidar K (2016). Asian Pac J Cancer Prev.

[24] Bandaru S (2016). Curr Pharm Des.

[25] Praseetha S (2016). Asian Pac J Cancer Prev.

[26] Gutlapalli VR (2015). Bioinformation.

[27] Gudala S (2015). Asian Pac J Cancer Prev.

[28] Dunna NR (2015). Asian Pac J Cancer Prev.

[29] Babitha PP (2015). Bioinformation.

[30] Nasr AB (2015). Bioinformation.

[31] Yadav M (2009). International Journal of Bioinformatics Research.

[32] Sahila MM (2015). Bioinformation.

[33] Bandaru S (2015). Asian Pac J Cancer Prev.

[34] Shaheen U (2015). Bioinformation.

[35] Kelotra A (2014). Bioinformation.

[36] Nayarisseri A (2013). J Pharm Res.

[37] Akare UR (2014). Bioinformation.

[38] Sinha C (2015). Curr Top Med Chem.

[39] Bandaru S (2015). Curr Top Med Chem.

[40] Dunna NR (2015). Curr Top Med Chem.

[41] Nayarisseri A (2015). Curr Top Med Chem.

[42] Kelotra S (2014). Asian Pac J Cancer Prev.

[43] Bandaru S (2014). Bioinformation.

[44] Sinha C (2014). Bioinformation.

[45] Tabassum A (2014). Interdiscip Sci.

[46] Nayarisseri A (2013). Interdiscip Sci.

[47] Bandaru S (2013). Curr Top Med Chem.

[48] Rao DM (2010). International Journal of BioinformaticsResearch.

